# Low carbohydrate, high fat diet impairs exercise economy and negates the performance benefit from intensified training in elite race walkers

**DOI:** 10.1113/JP273230

**Published:** 2017-02-14

**Authors:** Louise M. Burke, Megan L. Ross, Laura A. Garvican‐Lewis, Marijke Welvaert, Ida A. Heikura, Sara G. Forbes, Joanne G. Mirtschin, Louise E. Cato, Nicki Strobel, Avish P. Sharma, John A. Hawley

**Affiliations:** ^1^ Sports NutritionAustralian Institute of SportCanberraAustralia2616; ^2^ Mary MacKillop Institute for Health ResearchAustralian Catholic UniversityMelbourneAustralia3000; ^3^ University of Canberra Research Institute for Sport and ExerciseCanberraAustralia2617; ^4^ Innovation, Research and DevelopmentAustralian Institute of SportCanberraAustralia2616; ^5^ University College ZealandAnkerhusDenmark; ^6^ PhysiologyAustralian Institute of SportCanberraAustralia2616; ^7^ Research Institute for Sport and Exercise SciencesLiverpool John Moores UniversityLiverpoolUK

**Keywords:** athletic performance, ketogenic diet, sports nutrition

## Abstract

**Key points:**

- Three weeks of intensified training and mild energy deficit in elite race walkers increases peak aerobic capacity independent of dietary support.
- Adaptation to a ketogenic low carbohydrate, high fat (LCHF) diet markedly increases rates of whole‐body fat oxidation during exercise in race walkers over a range of exercise intensities.
- The increased rates of fat oxidation result in reduced economy (increased oxygen demand for a given speed) at velocities that translate to real‐life race performance in elite race walkers.
- In contrast to training with diets providing chronic or periodised high carbohydrate availability, adaptation to an LCHF diet impairs performance in elite endurance athletes despite a significant improvement in peak aerobic capacity.

**Abstract:**

We investigated the effects of adaptation to a ketogenic low carbohydrate (CHO), high fat diet (LCHF) during 3 weeks of intensified training on metabolism and performance of world‐class endurance athletes. We controlled three isoenergetic diets in elite race walkers: high CHO availability (g kg^−1^ day^−1^: 8.6 CHO, 2.1 protein, 1.2 fat) consumed before, during and after training (HCHO, *n* = 9); identical macronutrient intake, periodised within or between days to alternate between low and high CHO availability (PCHO, *n* = 10); LCHF (< 50 g day^−1^ CHO; 78% energy as fat; 2.1 g kg^−1^ day^−1^ protein; LCHF, *n* = 10). Post‐intervention, V˙O2 peak  during race walking increased in all groups (*P* < 0.001, 90% CI: 2.55, 5.20%). LCHF was associated with markedly increased rates of whole‐body fat oxidation, attaining peak rates of 1.57 ± 0.32 g min^−1^ during 2 h of walking at ∼80% V˙O2 peak . However, LCHF also increased the oxygen (O_2_) cost of race walking at velocities relevant to real‐life race performance: O_2_ uptake (expressed as a percentage of new V˙O2 peak ) at a speed approximating 20 km race pace was reduced in HCHO and PCHO (90% CI: −7.047, −2.55 and −5.18, −0.86, respectively), but was maintained at pre‐intervention levels in LCHF. HCHO and PCHO groups improved times for 10 km race walk: 6.6% (90% CI: 4.1, 9.1%) and 5.3% (3.4, 7.2%), with no improvement (−1.6% (−8.5, 5.3%)) for the LCHF group. In contrast to training with diets providing chronic or periodised high‐CHO availability, and despite a significant improvement in V˙O2 peak , adaptation to the topical LCHF diet negated performance benefits in elite endurance athletes, in part due to reduced exercise economy.

## Key points

- Three weeks of intensified training and mild energy deficit in elite race walkers increases peak aerobic capacity independent of dietary support.
- Adaptation to a ketogenic low carbohydrate, high fat (LCHF) diet markedly increases rates of whole‐body fat oxidation during exercise in race walkers over a range of exercise intensities.
- The increased rates of fat oxidation result in reduced economy (increased oxygen demand for a given speed) at velocities that translate to real‐life race performance in elite race walkers.
- In contrast to training with diets providing chronic or periodised high carbohydrate availability, adaptation to an LCHF diet impairs performance in elite endurance athletes despite a significant improvement in peak aerobic capacity.

**Abbreviations**
BM: body mass
CHO: carbohydrate
CI: confidence interval
FADH_2_: flavin adenone dinucleotide
HCHO: high carbohydrate availability
HR: heart rate
IAAF: International Association of Athletics Federations
LCHF: low carbohydrate, high fat
NADH: nicotinamide adenine dinucleotide
PCHO: periodised carbohydrate availability
RER: respiratory exchange ratio
RPE: rating of perceived exertion
${\dot{V}}_{O_{2}}$: volume of oxygen uptake
${\dot{V}}_{CO_{2}}$: volume of carbon dioxide production
V˙O2 peak : peak aerobic capacity

## Introduction

During continuous exercise lasting more than a few minutes duration, skeletal muscle is fuelled by both intra‐ and extramuscular carbohydrate (CHO) and lipid substrates, with only a minor contribution from amino acids (for review see Hawley *et al*. 2015). These observations were made over a century ago by Zuntz & Schumburg (1901) who manipulated the proportions of fat and CHO in the diet for several days and showed changes in the non‐protein respiratory exchange ratio (RER) during subsequent submaximal exercise. These experiments identified a potential benefit for CHO as a substrate for muscle metabolism by virtue of an ∼8% higher energy yield per litre of oxygen (O_2_) consumed when CHO was the primary fuel oxidised. Krogh & Lindhard (1920) confirmed the findings of Zuntz & Schumburg (1901) and reported a 5.5% greater energy yield (per litre of O_2_ consumption) during laboratory cycling with CHO rather than fat dependence. These workers also reported that subjects perceived a fixed, submaximal exercise task to be easier after several days of consuming a high CHO diet than when the preceding diet was CHO‐restricted and high in fat.

The application of surgical techniques to exercise biochemistry in the 1960s (Bergstrom & Hultman, 1966) made it possible to obtain direct measurement of substrate storage and utilisation from skeletal muscle biopsy samples and precipitated a more sophisticated understanding of the role of dietary CHO in determining exercise capacity (Bergstrom *et al*. 1967) and endurance performance (Karlsson & Saltin, 1971). Largely as a result of these studies, sport nutrition guidelines in the early 1990s promoted a high CHO diet, particularly around exercise sessions, to maximise muscle glycogen stores, and exogenous CHO fuels for both training and competition (Coyle, 1991). However, recent guidelines (Burke *et al*. 2004, 2011; Thomas *et al*. 2016) have evolved to acknowledge that a universal recommendation of absolute CHO intake is unsuitable because (1) the highly variable nature of exercise/sporting activities dictates that the quantity and timing of dietary CHO should be prescribed according to the anticipated fuel needs of exercise (‘CHO availability’), (2) high CHO availability is beneficial for competition sessions and for specific training sessions where high intensity exercise and performance outcomes are required, but it may be unnecessary when sustained intense exercise is less important, and (3) the quantity and timing of CHO intake should be personalised to the athlete and periodised within the various micro‐ and macro‐cycles of training and competition requirements. Furthermore, there is recognition that a periodised programme that includes some training sessions deliberately undertaken with low endogenous and/or exogenous CHO availability (‘train low’; Hawley & Burke, 2010; Bartlett *et al*. 2015) or a delay in replacing muscle glycogen after a session (‘sleep low’) may promote greater cellular adaptations to training (Lane *et al*. 2015) and enhance performance (Marquet *et al*. 2016) to a greater magnitude than undertaking all sessions with high CHO availability.

An alternative view on the optimal fuel support for sports performance, however, is to maximise the contribution of fat as a substrate for exercising muscle by following a low CHO, high fat diet (Noakes *et al*. 2014; Volek *et al*. 2015). A key argument in favour of this notion is that even the leanest athlete has an abundance of endogenous lipid stores in comparison to limited CHO reserves. Diets high in fat upregulate the release, transport, uptake and utilisation of fat in the muscle, even in endurance athletes whose training would have been expected to maximise such adaptations (Spriet, 2014; Burke, 2015). This concept has been explored in trained individuals in various formats over the past 40 years. Strategies have included chronic exposure to either a ketogenic (< 20 g day^−1^ CHO), high fat (80% of energy) diet (Phinney *et al*. 1983) or a restricted CHO (15–20% of energy), high fat (60–65% of energy) diet (Lambert *et al*. 1994; Goedecke *et al*. 1999) as well as short‐term adaptation to a high fat diet and 1 day of high CHO availability (Burke *et al*. 2000, 2002; Carey *et al*. 2001; Havemann *et al*. 2006). Although these iterations of high(er) fat, low(er) CHO diets result in increased rates of fat oxidation during exercise of varying intensities, evidence that this substrate shift translates to a clear enhancement of sports performance in athletic populations is lacking (Burke & Kiens, 2006; Burke, 2015).

Despite this, there has been a resurgence of interest in the published literature and social media (Burke, 2015) in a specific application of the high fat diet for athletes; the chronic consumption of a very low (< 50 g day^−1^) CHO, moderate protein, high fat (75‐80% of energy) diet. The dual intent of this strategy, termed LCHF or the ‘keto‐diet’, is to increase the utilisation of fat as a muscle fuel and to expose the body to high levels of circulating ketones (Noakes *et al*. 2014; Volek *et al*. 2015). To date, the only published investigations of this diet in endurance athletes are a single intervention study (Phinney *et al*. 1983) and two recent cross‐sectional studies comparing ultra‐endurance runners/triathletes who have chosen this eating style with similar athletes following higher CHO diets (Volek *et al*. 2016; Webster *et al*. 2016). Since none of these investigations measured the effects of LCHF on sports performance or were undertaken using exercise of an intensity that is relevant to the majority of competitive endurance athletes, there is a need to systematically investigate claims that LCHF enhances endurance performance (Burke, 2015). Accordingly, we determined the effects of a 3‐week adaptation to an LCHF diet during a period of intensified training on exercise metabolism and performance of world‐class endurance athletes. Comparisons of interest included both the traditional sports nutrition guidelines of training with sustained high CHO availability and the contemporary ideas around periodised CHO availability. We hypothesised that an LCHF diet would result in increased rates of fat utilisation during exercise, but would impair exercise economy and fail to translate into a performance benefit.

## Methods

### Ethical approval

This study conformed to the standards set by the *Declaration of Helsinki* and was approved by the Ethics Committee of the Australian Institute of Sport (AIS, no. 20150802). After comprehensive details of the study protocol were explained to the subjects orally and in writing, all participants provided their written informed consent.

### Overview of study design

**Figure 1:**
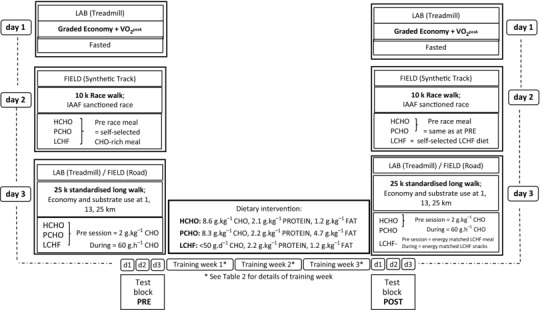
Overview of 3‐week training–diet intervention and testing blocks in elite race walkers undertaking high carbohydrate availability (HCHO, *n* = 9), periodised carbohydrate availability (PCHO, *n* = 10) or ketogenic low carbohydrate, high fat (LCHF, *n* = 10) diet

This study was conducted over two separate training camps in November 2015 (*n* = 11) and January 2016 (*n* = 19), which represented baseline preparation for the 2016 International Association of Athletics Federations (IAAF) race‐walking season and the qualification period for the 2016 Olympic Games. One subject was injured at the onset of the first camp and could not complete the required training and so was removed from the study. Overall, 21 male race walkers participated in the study, with eight athletes completing two camps, providing a total of 29 data sets. The design and implementation of the study involved a pragmatic blend of rigorous scientific control and research methodology with real‐world allowances needed to accommodate elite athlete populations. The study took place at the Australian Institute of Sport (AIS) with participants living in athlete residences and undertaking all meals and training sessions under supervision. The study consisted of a 3‐week structured programme of intensified training with a testing block carried out immediately before and afterwards (see Fig. 1).

### Participants and allocation to interventions

We established an *a priori* sample size of 10 subjects per treatment group, based on previous experience of detecting clear improvements in physiological characteristics and performance over a 3‐ to 4‐week training period in studies involving 8–10 subjects (Lindsay *et al*. 1996; Cox *et al*. 2010) as well as the resources available for rigorous control of the intervention and outcome monitoring. We recruited highly competitive male race walkers, based on performances ranked by the IAAF, via word of mouth and targeted invitations from a key athlete and coach with whom the study was planned. A total of 21 participants all of whom had had international race experience were accepted. The cohort ranged from world class athletes (e.g. a seven‐time Olympic and World Championship medallist from 2008–2016, a 2015 World Championship medallist, members of the second placed team from the 2016 World Walking Cup event and holders of national and continental records) to highly trained athletes (a junior international race walker, training partners of world class athletes). Of the 29 data sets collected in this study, 22 involved race walkers who were selected for at least one of the two major events for the 2016 calendar: the IAAF Race Walking Team Championships and the Summer Olympic Games.

The study intervention involved three different approaches to dietary support for the intensified training programme: high CHO availability (HCHO), periodised CHO availability (PCHO) and low CHO, high fat (LCHF). Our goal was to ensure that participants perceived they would receive benefits for their race preparation, while matching groups for key characteristics (age, current aerobic capacity, personal best times for the 20 km race walk event and training history and intended training load). Given the potential for a placebo effect, the departure of at least one of the treatments from current sports nutrition guidelines and the pragmatism required to work with world class athletes in preparation for key events, we devised a method for allocating participants to the treatments in this study. Prior to their arrival to the study camps, participants were educated about the benefits and limitations of the different dietary treatments and asked to nominate their preference(s) for, or non‐acceptance of, each of these interventions. We were able to allocate the race walkers to a preferred treatment for each of the study camps, including the preferred order of treatment for those who participated in two camps, while achieving suitable matching of groups based on age, body mass, aerobic capacity and personal best for the 20 km race walking event (Table 1), and matching of diet allocations between the two camps. Residual differences between groups and camps, and the involvement of both single and dual participation were included in the statistical analyses.

**Table 1:** Characteristics of elite race walkers (*n* = 21) in training and diet intervention study (*n* = 29 cases)

	High carbohydrate (HCHO)	Periodised carbohydrate (PCHO)	Low carbohydrate, high Fat (LCHF)
Sample size (number who undertook second trial)	*n* = 9 (*n* = 5: PCHO = 2; LCHF = 3)	*n* = 10 (*n* = 5: HCHO = 2; LCHF = 3)	*n* = 10 (*n* = 6: PCHO = 3; HCHO = 3)
Age (years)	25.4 (4.0)	27.4 (4.6)	28.3 (3.5)
BM (kg)	63.9 (8.5)	66.8 (6.8)	66.6 (7.2)
Baseline V˙O2 peak (ml kg^−1^ min^−1^) during race walking test	61.6 (6.8)	64.6 (5.3)	66.3 (4.8)
Personal Best in 10 km race walk (min:s)	41:20 (0:43)	40:31 (1:45)	40:43 (1:16)
Personal Best in 20 km race walk (min:s)*	85:14 (3:46)	82:51 (4:18)	82:34 (2:50)

Data are mean (SD). ^*^Qualifying standard for 20 km race walking event at 2016 Summer Olympic Games = 84 min.

### Training intervention

All participants undertook 3 weeks of intensified training incorporating race walking, resistance training and cross‐training (running, cycling or swimming). Training sessions were undertaken as a group and were monitored by the research team as well as recorded by participants in a daily log. The template for the weekly training programme was developed in collaboration with a world class race walker and several coaches to blend the typical intensified training practices of competitive walkers with opportunities to implement the desired dietary intervention. Table 1 details the weekly programme, noting six mandatory sessions that were undertaken under standardised conditions with external monitoring. The remaining sessions were undertaken according to the preference of the individual athlete and noted in the participant's training logs.

### Dietary intervention

All foods and fluids consumed during this study were provided by the AIS Sports Nutrition team, and recorded. Menu construction and the preparation of meals and snacks were undertaken by a professional chef, food service dietitians and sports dietitians. Meal plans were individually developed for each athlete to integrate personal food preferences and nutrition requirements within his allocated dietary treatment. Meals were eaten in a separate dining area in a group setting with individualised meals being served for each athlete according to their meal plans. During each meal service, the weight of each food item was recorded using calibrated scales (accurate to 2 g). Individualised snacks were provided for intake between meals and before or during training sessions, with the requirement for their consumption to be cross‐checked at the next meal. A range of ‘free foods and drinks’ (foods with low energy such as fruits and vegetables, tea or coffee, water and artificially sweetened beverages) were provided in the participants’ living area with a checklist to allow each participant to report on his day's intake at the first meal of the following day. Nutrition support during longer training sessions and after key sessions was provided at the training site by members of the research team and intake was recorded. Full details of the menu plans and study approach are provided elsewhere (Mirtschin *et al*. in review). Compliance to the dietary prescription and reporting requirements was checked daily.

As a primary goal of the study was to evaluate three different dietary treatments without any interference due to large or different changes in body composition, energy intake for each athlete was set at 40 kcal (kg lean body mass (BM))^−1^ day^−1^. A loss of fat mass of ∼1–1.5 kg over the 4‐week intervention was permitted and each athlete could request additional food at meals or from designated snacks and ‘free snacks’ according to hunger, increases in training load from the baseline information or large fluctuations in BM above that expected with the glycogen or fluid shifts associated with the LCHF. When such variations occurred, they were achieved by following the macronutrient composition of the treatment diet and noted in the actual food consumed.

The three dietary treatments investigated in the study are summarised below (further detail provided in Table 2):

1. High CHO availability (HCHO). Overall macronutrient composition 60–65% of energy from CHO, 15–20% protein, 20% fat; similar daily CHO intake, with CHO consumed before, during and after training sessions. The diet represents sports nutrition guidelines from the 1990s (Coyle, 1991).
2. Periodised CHO availability (PCHO). Same overall macronutrient composition as HCHO, but spread differently between and within days according to fuel needs of training as well as an integration of some training sessions with high CHO availability (high muscle glycogen, CHO feeding during session) and others with low CHO availability (low pre‐exercise glycogen, overnight fasted or delayed post‐session refuelling). This strategy represents current guidelines (Thomas *et al*. 2016) and evolving evidence around benefits of strategic training with low CHO availability (Bartlett *et al*. 2015; Marquet *et al*. 2016).
3. Low‐CHO, high fat diet (LCHF): 75–80% fat, 15–20% protein, < 50g day^−1^ CHO. A ketogenic diet following the guidelines previously reported (Volek & Phinney, 2012) and utilised in a study undertaken with endurance cyclists (Phinney *et al*. 1983).

**Table 2:** Overview of weekly training‐diet intervention involving high carbohydrate (CHO) availability (HCHO), periodised CHO availability (PCHO) or low CHO high fat (LCHF) diets in elite race walkers (*n* = 29)

Day	Diet	Monday	Tuesday	Wednesday	Thursday	Friday	Saturday	Sunday
AM training		Easy 10 km	Easy 10–15 km*, gym	Long* (20–40 km)	Easy 10–15 km*, gym	Hill session*	Long* (25–40 km)	Easy 10km/nil
Dietary treatment around training session	HCHO (*n* = 9)	CHO pre, during, post	CHO pre, during, post	CHO pre, during, post	CHO pre, during, post	CHO pre, during, post	CHO pre, during, post	CHO pre, during, post
	PCHO (*n* = 10)	Fasted training; CHO post	Fasted training + low glycogen (Train low†); CHO post	CHO pre, during, post	Fasted training; CHO post	CHO pre + during; Nil CHO post (Sleep low†)	CHO pre, during, post	Fasted training; CHO post
	LCHF (*n* = 10)	Minimal CHO; high fat	Minimal CHO; high fat	Minimal CHO; high fat	Minimal CHO; high fat	Minimal CHO; high fat	Minimal CHO; high fat	Minimal CHO; high fat
PM training		Interval session*	Easy 10 km	Easy 10 km/nil	10–15 km	Easy 10–15 km*	Easy 10 km/ nil	Easy/nil
Dietary treatment around training session	HCHO (*n* = 9)	CHO pre, during, post	CHO pre, during, post	CHO pre, during, post	CHO pre, during, post	CHO pre, during, post	CHO pre, during, post	CHO pre, during, post
	PCHO (*n* = 10)	CHO pre, during; minimal CHO post (Sleep low†)	CHO pre, during, post	Fasted training CHO post	CHO pre, during, post	Fasted training + low glycogen (Train low†); CHO post	CHO pre, during, post	CHO pre, during, post
	LCHF (*n* = 10)	Minimal CHO; high fat	Minimal CHO; high fat	Minimal CHO; high fat	Minimal CHO; high fat	Minimal CHO; high fat	Minimal CHO; high fat	Minimal CHO; high fat

^*^Compulsory key training session. ^†^CHO periodisation strategy reviewed by Hawley & Burke (2010).

### Test block

Immediately before and after the 3‐week training intervention, participants completed a 3‐day test block (Fig. 1). Components of the testing are described below and involved laboratory testing, a field test of performance, and a hybrid session of laboratory and field exercise. Each test was undertaken in the same order, and conditions were standardised for each participant

#### V˙O2 peak  and walking economy

On Day 1 of the testing block, participants completed a treadmill test to assess economy and V˙O2 peak  while race walking. This test was undertaken in a fasted, rested state for both pre‐treatment and post‐treatment tests. Walking economy was assessed during four submaximal stages, each of 4 min duration, increasing in speed by 1 km h^−1^ each stage. Depending on each walker's capability, the speed of the first stage was 11 or 12 km h^−1^ and increased to 14–15 km h^−1^ for the last stage. Typically, the second and fourth stage of this test corresponded to an individual's race pace for the 50‐ and 20‐km race walking events, respectively. Each 4 min stage was followed by 1 min of rest during which the treadmill was stopped to allow the collection of capillary blood samples (finger tip) and ratings of perceived exertion (6–20, Borg Scale). Heart rate (HR) was measured continuously during the test (Polar Heart Rate Monitor, Polar Electro, Kempele, Finland). Expired gas was collected and analysed using a custom‐built indirect calorimetry system described previously (Robertson *et al*. 2010), with the final 60 s of gas collection accepted as steady state and used to calculate RER and O_2_ uptake.

On completion of the final submaximal walking stage, subjects rested for 5 min before completing a ramp (speed and then gradient) test to volitional fatigue. Treadmill speed recommenced at the first stage of the economy test and was increased by 0.5 km h^−1^ every 30 s until the speed corresponding to the final submaximal stage was reached (14 or 15 km h^−1^). From then on, the treadmill gradient was increased by 0.5% every 30 s until exhaustion (defined as an inability of the athlete to keep pace with the treadmill). HR measurements and capillary blood samples were collected 1 min after completion of the test.

#### 10 km race

On Day 2 of the testing block, the race walkers competed in an IAAF‐sanctioned 10 km race held on a 400 m outdoor synthetic athletics track (Canberra, ACT, Australia). To provide incentive for a maximal effort, prize money was awarded to place getters as well as those athletes who achieved the highest percentage of their 20 km walking personal best when the times of the two races (pre‐ and post‐treatment) were combined. Each race commenced at 0830 h and was conducted under IAAF rules, which involved officiating by technical judges, invitation for participation by competitors external to the study, a feed zone allowing water intake on the outside lanes of the track in hot conditions, and photo‐finish electronic timing. Photo‐finish timing was used to provide official race times. Capillary blood samples were collected immediately before the start of the race and as each competitor completed the 10 km distance.

For the pre‐treatment testing block, participants were permitted to consume their habitual pre‐race diet for the 24 h prior to the race and their pre‐race (morning) meal provided it was documented accurately. Use of performance supplements (e.g. caffeine) was discussed with each participant prior to the first race; permission was provided when it didn't interfere with the treatment diet, was documented, and was repeated for the post‐treatment race. For the post‐treatment race, participants followed the diet consistent with their treatment for the 24 h pre‐race period and consumed a pre‐race meal according to their dietary intervention (e.g. high in CHO or high in fat).

#### Standardised 25 km long walk

On the third day of the testing block, subjects completed a 25 km walk, 2 h after consuming a standardised breakfast. The training session was conducted as a hybrid laboratory–field test, with 0–1, 12–13 and 24–25 km being undertaken on a treadmill in the laboratory and the remainder of the walk completed outdoors on a loop course (∼5 km) that included two aid stations to allow nutritional support to be received each ∼2 km as occurs in IAAF events. Subjects completed the treadmill portions of the walk at the speed corresponding to the second stage of the submaximal walking test (12 or 13 km h^−1^), which approximated their 50‐km race pace. Expired gas was collected during each treadmill segment for assessment of RER to determine rates of substrate oxidation, and O_2_ uptake. Capillary blood was collected for measurement of glucose, lactate and ketone concentrations immediately prior to beginning the session, and upon completion of each treadmill segment. HR and rating of perceived exertion (RPE) were assessed at the end of each treadmill section. Nutritional support for this session is summarised in Fig. 1. In the pre‐treatment trial, all subjects received a standardised CHO‐rich breakfast providing 2 g kg^−1^ CHO; this same meal was provided to the HCHO and PCHO group participants in the post‐treatment trial. Meanwhile, in the post‐treatment trial, the LCHF group received an isocaloric meal that was high fat, low CHO. During the walk, handlers provided fluids and sports food choices that had previously been discussed with each participant to mimic race feeding practices. Participants were offered sports drink, sports gels and confectionery items to achieve an hourly intake of ∼600 ml of fluid and ∼60 g CHO in the pre‐treatment. In the post‐treatment trial, the HCHO and PCHO participants were provided with the same choices as consumed in the first trial. Meanwhile, in the post‐treatment trial, the LCHF received non‐caloric fluid (water or artificially sweetened drinks) and fat‐rich snacks (cheese and cake/cookies made from high fat ingredients) to match the energy intake from the first trial.

#### Blood metabolites

Capillary blood samples were used for this portion of the study to allow standardised collection of samples in both laboratory and field conditions where metabolites were to be assessed over the entire 4‐week intervention. Finger‐tip samples were collected and immediately processed for measurement of blood lactate (Lactate Pro 2, Akray, Japan), ketones (β‐hydroxybutyrate; FreeStyle Optium Neo, Abbott Diabetes Care, Victoria, Australia) and glucose (FreeStyle Optium Neo, Abbott Diabetes Care, Victoria, Australia) concentrations. To counter any individual differences in the accuracy of these portable analysers, each participant was assigned to a specific device for the duration of their involvement in the study.

#### Calculation of respiratory exchange ratio and substrate oxidation data

Respiratory exchange ratio (RER) was calculated from steady‐state expired gases collected over 1 min periods during the economy test, maximal aerobic capacity protocol and portions of the 25 km standardised walk. During the 25 km standardised walk, rates of CHO and fat oxidation (g min^−1^) were calculated from ${\dot{V}}_{CO_{2}}$ and ${\dot{V}}_{O_{2}}$ values using non‐protein RER values (Peronnet & Massicotte, 1991). These equations are based on the premise that ${\dot{V}}_{O_{2}}$ and ${\dot{V}}_{CO_{2}}$ accurately reflect tissue O_2_ consumption and CO_2_ production, and that indirect calorimetry is a valid method for quantifying rates of substrate oxidation in well‐trained subjects during strenuous exercise of up to ∼85% of V˙O2 peak  (Romijn *et al*. 1992). We did not correct our calculations for the contribution of ketone oxidation to substrate use in the LCHF trials as we wished to directly compare our findings with other recent reports of substrate utilisation in ultra‐endurance athletes who chronically consume LCHF diets (Volek *et al*. 2016; Webster *et al*. 2016). However, we acknowledge that there may be a small (but systematic) error in the use of conventional equations to calculate fat and CHO oxidation from gas exchange information (Frayn, 1983).

### Statistical analysis

Statistical analyses were carried out using a general linear mixed model using the R package lme4 (Bates *et al*. 2015; R Core Team, 2016) allowing for the unbalanced design (i.e. cross‐over of some subjects between camps and unequal group sizes for diet intervention) and the identified heterogeneity in the individual data (Jennrich & Schluchter, 1986). Each dependent variable was included in a mixed model with fixed effect for the diet intervention, the race and the camp, including all two‐way and three‐way interactions. In the random effects structure of the model, we included a crossed random effect for race and camp to account for heterogeneity in race conditions, as well as a random intercept for the athletes to account for the dependence in the data. Non‐significant higher order interactions were dropped from the model for ease of interpretation. The normality assumption of the linear mixed model was assessed visually using QQ‐plots of the model residuals. No obvious deviations of normality were detected. Tests for statistical significance of the fixed effects were performed using Type II Wald tests with Kenward–Roger degrees of freedom. Ninety per cent confidence intervals (CI) for the fixed effects were calculated using parametric bootstrap.

## Results

### Diet and training compliance

All participants demonstrated compliance to their assigned dietary treatment and the monitoring of their food intake and training sessions. Table 3 summarises the results of the assessment of actual dietary intake, with the summary of mean daily intakes allowing the display of overall differences between diets. As intended, energy (kJ kg^−1^ day^−1^) and protein (g kg^−1^ day^−1^ and % energy) intake did not differ between dietary treatments. However, daily fat (g kg^−1^ day^−1^ and % of energy) and CHO (g kg^−1^ day^−1^ and % energy) intakes were significantly skewed between the CHO‐rich diets (HCHO and PCHO) and the LCHF diet. Although mean daily intake of CHO was similar between the HCHO and PCHO diets, the breakdown associated with specific training days partially illustrates the difference in its spread, due to the timing of intake around training sessions designated as high and low CHO availability. The deliberate but small energy deficit permitted over the 4 weeks of intervention and testing resulted in a similar change of body composition across groups (mean loss of ∼1.4 kg of body fat; not significant). While this would have contributed towards an improvement in aerobic capacity and walking economy (relative to BM) across all groups, we feel that energy availability was preserved and standardised such that it did not interfere with training adaptations or favour one group over another.

**Table 3:** Summary of actual dietary intake by elite race walkers (*n* = 29) in training‐diet intervention involving high carbohydrate availability (HCHO), periodised CHO availability (PCHO) or low CHO high fat (LCHF) diets; data are mean (SD)

Diet	Nutrient	Mean daily intake	Mean intake Monday (g kg^−1^ day^−1^)	Mean intake Tuesday (g kg^−1^ day^−1^)	Mean intake Wednesday (g kg^−1^ day^−1^)	Mean intake Thursday (g kg^−1^ day^−1^)	Mean intake Friday (g kg^−1^ day^−1^)	Mean intake Saturday (g kg^−1^ day^−1^)	Mean intake Sunday (g kg^−1^ day^−1^)
HCHO (*n* = 9) Daily energy intake: 14.73 MJ 231 kJ kg^−1^	Protein	138 g 2.1 g kg^−1^ 16% energy	2.1 (0.1)	2.3 (0.2)	2.2 (0.2)	2.3 (0.3)	2.1 (0.1)	2.1 (0.1)	2.1 (0.3)
	CHO	549 g	8.6 (0.4)	8.7 (0.8)	8.5 (0.8)	8.8 (0.7)	8.4 (0.7)	8.8 (0.8)	8.5 (1.2)
		8.6 g kg^−1^							
		60% energy							
	Fat	77 g 1.2 g kg^−1^ 20% energy	1.2 (0.1)	1.3 (0.2)	1.2 (0.1)	1.3 (0.1)	1.1 (0.2)	1.2 (0.1)	1.2 (0.2)
PCHO (*n* = 10) Daily energy intake: 14.89 MJ 226 kJ kg^−1^	Protein	144 g 2.2 g kg^−1^ 17% energy	2.2 (0.2)	2.4 (0.3)	2.1 (0.1)	2.4 (0.4)	2.3 (0.1)	2.2 (0.2)	2.2 (0.1)
	CHO	547 g	8.6 (1.0)	7.0 (1.2)*	9.1 (0.6)	8.3 (0.9)	6.0 (0.6)†	9.5 (0.8)	9.0 (0.8)
		8.3 g kg^−1^							
		60% energy							
	Fat	79 g 1.2 g kg^−1^ 20% energy	1.2 (0.1)	1.4 (0.2)	1.2 (0.1)	1.4 (0.4)	1.2 (0.2)	1.2 (0.1)	1.2 (0.1)
LCHF (*n* = 10) Daily energy intake: 14.90 MJ 223 kJ kg^−1^	Protein	144 g 2.2 g kg^−1^ 17% energy	2.1 (0.1)	2.1 (0.1)	2.3 (0.2)	2.1 (0.1)	2.1 (0.1)	2.2 (0.2)	2.2 (0.2)
	CHO	33 g	0.6 (0.1)	0.4 (0.1)	0.5 (0.1)	0.5 (0.1)	0.5 (0.1)	0.5 (0.2)	0.5 (0.1)
		0.5 g kg^−1^							
		3.5% energy							
	Fat	312 g 4.7 ^−1^ 78% energy	4.7 (0.4)	4.7 (0.5)	4.7 (0.4)	4.6 (0.4)	4.5 (0.3)	5.1 (0.8)	4.5 (0.3)

^*^Carbohydrate (CHO) intake restricted from after PM training until after next AM training. ^†^CHO intake restricted from after AM training until after PM training.

Participants completed the 3‐week training intervention, with weekly volume totals showing a similar overall training commitment between groups, although participants in the LCHF group experienced greater perception of effort throughout, often experiencing substantial hardship or inability to complete sessions as planned. Analysis of training quality and general well‐being during this training block are detailed elsewhere (Ross *et al*. in review). Mean race walking mileage for the HCHO group was 121, 127 and 103 km and 351 (30) km week^−1^ for weeks 1–3 and total training, respectively. Meanwhile, training loads for PCHO were 137, 132 and 107 km week^−1^ with a total of 377 (43) km, while the LCHF group completed 112, 122 and 97 km week^−1^ for a total of 331 (56) km.

### V˙O2 peak  and economy testing

**Figure 2:**
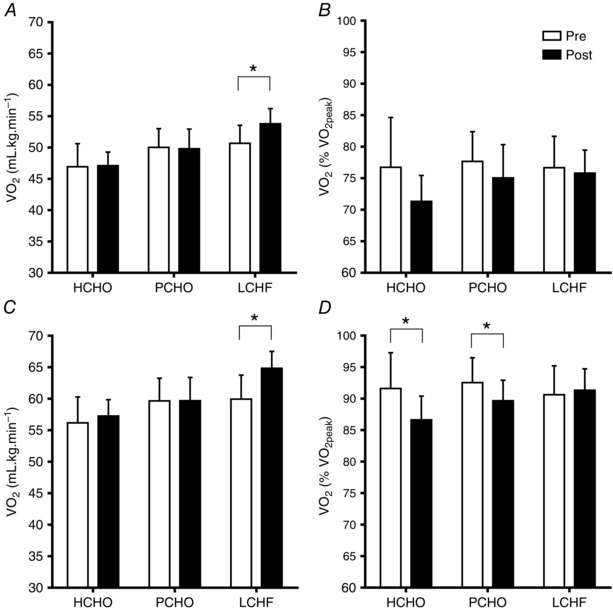
Oxygen uptake during graded economy test at second stage approximating 50 km race speed (*A*, ml kg^−1^ min^−1^ and *B*, % V˙O2 peak ) and fourth stage approximating 20 km race speed (*C*, ml kg^−1^ min^−1^ and *D*, % V˙O2 peak ) in elite race walkers pre‐ and post‐3 weeks of intensified training and high carbohydrate availability (HCHO, *n* = 9), periodised carbohydrate availability (PCHO, *n* = 10), or ketogenic low carbohydrate, high fat (LCHF, *n* = 10) diets ^*^Significantly different from pre‐treatment (*P* <  0.01).

**Figure 3:**
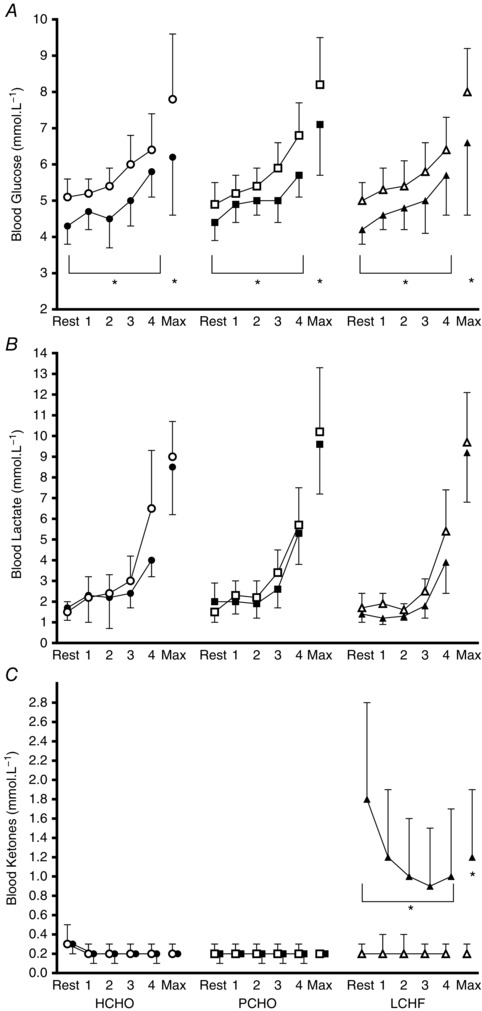
Blood metabolite concentrations (blood glucose (mmol l^−1^, *A*), blood lactate (mmol l^−1^, *B*) and blood ketones (β‐hydroxybutyrate, mmol l^−1^, *C*)) during graded economy test and test for peak aerobic capacity in elite race walkers pre‐ and post‐3 weeks of intensified training and high carbohydrate availability (HCHO, *n* = 9), periodised carbohydrate availability (PCHO, *n* = 10), or ketogenic low carbohydrate high fat (LCHF, *n* = 10) diets ^*^Significantly different from pre‐treatment (*P* <  0.01).

All race walkers participated in the graded economy and V˙O2 peak  test protocols. One walker (from the PCHO group) reported pain associated with a previous injury at the end of the fourth stage of the economy test and was withdrawn from the subsequent assessment of this variable. Therefore the sample sizes for these tests were 9, 10 and 10 for HCHO, PCHO and LCHF, respectively, for the economy testing phase, and 9, 9 and 10 for the V˙O2 peak  test. The results of these tests are summarised in Table 4 (BM, HR, RER, RPE and V˙O2 peak  data), Fig. 2 (O_2_ utilisation) and Fig. 3 (blood metabolites). At both testing times, there was an increase in RER (*F*(3, 197) = 255.44, *P *< 0.001), V˙O2 peak  (*F*(3, 193) = 1345.99, *P* < 0.001), HR (*F*(3, 188) = 519.27, *P* < 0.001) and RPE (*F*(3, 196) = 209.76, *P* < 0.001) associated with the increase in exercise intensities across all stages of the economy test.

**Table 4:** Results of testing on Day 1: graded economy test and maximal aerobic capacity before and after 3 week diet and training intervention in elite race walkers (*n* = 29)

		HCHO (*n* = 9)					PCHO (*n* = 10)					LCHF (*n* = 10)				
		S1	S2	S3	S4	Max	S1	S2	S3	S4	Max	S1	S2	S3	S4	Max
Variable	Time	*n* = 9				*n* = 9	*n* = 10				*n* = 9	*n* = 10				*n* = 10
Body mass (kg)	Pre	63.9 (8.5)					66.8 (6.8)					66.6 (7.2)				
	Post	63.3 (8.2)					65.2* (6.5)					64.8* (7.1)				
Respiratory	Pre	0.87	0.90	0.94	0.99	1.08	0.87	0.91	0.95	1.00	1.06	0.86	0.90	0.94	0.99	1.10
exchange ratio		(0.06)	(0.04)	(0.03)	(0.04)	(0.05)	(0.03)	(0.03)	(0.03)	(0.03)	(0.06)	(0.03)	(0.04)	(0.04)	(0.04)	(0.13)
	Post†	0.87	0.91	0.93	0.94	1.09	0.88	0.91	0.94	0.99	1.08	0.73	0.77	0.80	0.87	0.97*
		(0.02)	(0.02)	(0.02)	(0.3)	(0.03)	(0.04)	(0.03)	(0.04)	(0.05)	(0.05)	(0.02)	(0.02)	(0.03)	(0.03)	(0.05)
${\dot{V}}_{O_{2}}$ (l min^−1^)	Pre	2.66	3.02	3.34	3.61	3.93	3.05	3.34	3.69	3.98	4.37	3.04	3.38	3.71	4.00	4.47
		(0.53)	(0.61)	(0.64)	(0.65)	(0.74)	(0.36)	(0.39)	(0.41)	(0.44)	(0.53)	(0.43)	(0.45)	(0.50)	(0.58)	(0.64)
	Post	2.66	2.99	3.31	3.63	4.20*	2.87	3.25	3.57	3.89	4.41*	3.02	3.50	3.87	4.21	4.56*
		(0.48)	(0.47)	(0.48)	(0.65)	(0.66)	(0.31)	(0.35)	(0.39)	(0.44)	(0.44)	(0.61)	(0.48)	(0.55)	(0.59)	(0.70)
Heart rate (beats min^−1^)	Pre	154	167	179	187	197	148	159	170	177	187	148	163	174	182	191
		(15)	(12)	(11)	(10)	(8)	(10)	(6)	(5)	(4)	(5)	(8)	(9)	(8)	(10)	(5)
	Post‡	133	150	162	172	187*	130	146	157	167	179*	148	160	172	181	191
		(8)	(9)	(9)	(10)	(9)	(12)	(6)	(5)	(4)	(6)	(6)	(10)	(5)	(6)	(5)
RPE	Pre	9.5	11.4	13.6	16.3		10.2	11.7	13.4	15.1		9.7	11.7	13.4	15.8	
		(2.3)	(1.6)	(2.1)	(2.1)		(1.6)	(1.4)	(1.1)	(1.3)		(2.2)	(1.6)	(1.6)	(1.9)	
	Post‡	10.1	11.4	13.6	15.6		9.6	11.3	13.2	15.0		9.6	12.8	14.6	16.9	
		(1.8)	(1.3)	(1.7)	(1.7)		(2.1)	(1.4)	(1.4)	(1.7)		(3.3)	(1.0)	(0.8)	(1.1)	

Data are mean (SD), and statistical comparisons note differences across the intervention and between groups receiving high carbohydrate availability (HCHO), periodised CHO availability (PCHO) and low carbohydrate, high fat (LCHF) dietary treatments. ^*^Difference between pre‐ and post‐treatment values within the group (*P* ≤ 0.02). †Lower in post‐treatment trial across stages 1–4 than in pre‐treatment trial for LCHF (*P* ≤ 0.001). ‡Higher in post‐treatment trial across stages 1–4 than pre‐treatment trial for LCHF (*P* ≤ 0.01).

BM decreased over the 3 weeks of intensified training and mild energy deficit, with losses being greater in the LCHF and PCHO groups compared with HCHO (90% CI: −1.89, −0.44 and −1.58, −0.24, respectively). V˙O2 peak  during race walking increased in all three groups across the training programme (*F*(1, 30) = 25.52, *P* < 0.001, 90% CI: 2.55, 5.20). When expressed in ml kg^−1^ min^−1^, the changes from pre‐ to post‐treatment were as follows: HCHO: 61.6 (90% CI: 57.9, 65.1) to 66.2 (63.7, 68.8); PCHO: 64.9 (61.9, 67.9) to 67.0 (64.5, 69.6) and LCHF: 66.3 (63.9, 68.7) to 71.1 (69.3, 72.8). The peak aerobic capacity of the HCHO group was lower than for the other two groups both pre‐ and post‐treatment (*F*(2, 24) = 4.44, *P *≤ 0.02).

Across the graded economy tests, there were significant diet *vs*. test interactions for RPE and HR with the LCHF group displaying higher values in the post‐treatment trial (*F*(2, 197) = 4.52, *P* = 0.01 and *F*(2, 162) = 50.13, *P* < 0.001, respectively), indicating a higher metabolic cost and perceived effort with this treatment compared with baseline testing and the other diets (Table 4). There was a significant decrease in post‐treatment RER values in the LCHF group (*F*(2, 194) = 172.79, *P* < 0.001) compared with the pre‐treatment trial across the four economy stages. Differences in pre‐ to post‐treatment values for HCHO and PCHO groups were minor. Concomitant with the change in fuel utilisation, there was an increase in the post‐treatment values for absolute O_2_ cost of exercise (l min^−1^) across the four economy test stages in the LCHF group compared with HCHO (90% CI: 0.09, 0.20. By contrast, the absolute O_2_ cost of exercise across the economy test was reduced at the post‐treatment trial in PCHO compared with the HCHO group (−0.17, −0.05). Separate analysis of characteristics at the completion of the V˙O2 peak  test revealed an increase in absolute values of O_2_ utilisation (l min^−1^) across the treatment period in all trials (*F*(1, 31) = 5.79, *P* = 0.02). However, there were differences in RER and HR as a result of the dietary treatment: RER at the point of maximal aerobic capacity in the post‐treatment trial was lower than pre‐treatment in the LCHF group (*F*(2, 31) = 12.13, *P* < 0.001, 90% CI: −0.12,−0.06). In addition, HR at this same point in the post‐treatment trial was lower than in the pre‐treatment trial for the HCHO and PCHO groups (*F*(2, 29) = 18.15, *P* < 0.001, 90% CI: −10.35,−6.37 and −9.15, −5.04, respectively).

Figure 2 illustrates the changes in substrate utilisation and the O_2_ cost of exercise due to diet and/or training by focusing on stage 2 (Fig 2 *A* and *B*) and stage 4 (Fig 2 *C* and *D*) of the economy test (corresponding to the walking speeds of elite race walkers during a 50 km event and 20 km event respectively). At the second stage (12–13 km h^−1^), there was a significant increase in the relative O_2_ cost of exercise (ml kg^−1^ min^−1^) in the LCHF group in the post‐treatment trial, while this remained constant in the PCHO and HCHO groups (*F*(2, 31) = 5.91, *P* = 0.006). When expressed as a percentage of their (increased) V˙O2 peak , there was a significant reduction in O_2_ utilisation at second stage speed in the HCHO and PCHO groups at the post‐treatment trial (90% CI: −8.48, −2.19 and −5.55, 0.23, respectively). The interaction of diet and training in the LCHF group was such that there was no improvement in the fraction of V˙O2 peak  used to walk at the second stage speed across the intervention. The pattern was repeated in the results from the fourth stage (14–15 km h^−1^) of the economy test in stronger form: there was a significant increase in the relative O_2_ cost of exercise (ml kg^−1^ min^−1^) in the LCHF group in the post‐treatment trial, while this remained constant in the PCHO and HCHO group (*F*(2, 31) = 16.67, *P* < 0.001). When expressed as a percentage of the (increased) V˙O2 peak , O_2_ utilisation at the fourth stage speed was reduced in the HCHO and PCHO groups at the post‐treatment trial (90% CI: −7.047, −2.55 and −5.18, −0.86, respectively), but was maintained in the LCHF group.

Figure 3 summarises concentrations of metabolites (glucose, lactate and ketones) in capillary blood samples obtained during the economy test, representing resting values after an overnight fast, and concentrations at the end of each stage and at the conclusion of the maximal aerobic capacity component. Blood glucose concentrations increased from baseline values during exercise, with an increase in exercise intensity being associated with high blood glucose concentrations (Resting, Step 1 < Step 2 < Steps 3, 4; *F*(4, 253) = 70.75, *P* < 0.001). Across all dietary treatments, there was a decrease in blood glucose concentrations across the training intervention such that values in the post‐treatment trials were lower than in the pre‐treatment trial (*F*(1,254) = 125.65, *P* < 0.001). Blood lactate concentrations increased as exercise intensity was increased across all dietary treatments (Resting, Steps 1, 2 < Steps 3, 4; *F*(4, 254) = 24,93, *P* < 0.001), with similar patterns in both pre‐ and post‐treatment trials. Blood ketone levels were maintained at low concentrations across the economy test for both pre‐ and post‐treatment trials with the HCHO and PCHO groups. However, the post‐treatment trial in the LCHF group was associated with a significant increase in blood ketone concentrations compared with the pre‐treatment trial (*F*(8, 221) = 3.64, *P* < 0.001). Elevated blood ketone concentrations were found in the resting sample; these declined over the four economy stages but still remained elevated in comparison with the pre‐treatment trial (Fig. 3). Blood ketone concentrations were elevated in the post‐treatment trial in the LCHF group with no change in the other diet groups (*F*(2, 25) = 19.59, *P* < 0.001). Blood glucose concentrations were reduced in the post‐treatment trial in all groups (*F*(1, 24) = 17.26, *P* < 0.001), but there was no difference in blood lactate concentrations between pre‐ and post‐treatment trials for any group.

### 10 km race

The study produced 26 data sets in which a race walker competed in a 10 km race pre and post their allocated intervention. Data sets were not available for two participants who sustained soreness or injury that prevented them from competing in the appropriate race. Race data were excluded from one participant (PCHO) who violated the standardised training preparation by undertaking a very long walk (30 km) on the day before the race. Race performance data therefore represent *n* = 9, 8 and 9 for HCHO, PCHO and LCHF, respectively. The 10 km race walking events were held on the same synthetic outdoor athletics track and were standardised for time of the day; however, environmental conditions varied for each of the races. In camp 1, conditions at the commencement of Race 1 were 17°C, 85% relative humidity (RH) and wind speed 1–1.5 m s^–1^ with gusts up to 2.5 m s^–1^, with those of Race 2 being 20.5°C, 46% RH, 4 m s^–1^ with gusts to 8 m s^–1^. In camp 2, conditions at the commencement of Race 1 were 28°C, 55% RH and wind speed: 0 m s^–1^, with those of Race 2 being 18°C, 65% RH, and 0.6–1.5 m s^–1^ with gusts to 3 m s^–1^. Differences in the race conditions were accounted for in the statistical analyses of group results but interfere with an interpretation of the results of individual race walkers.

**Figure 4:**
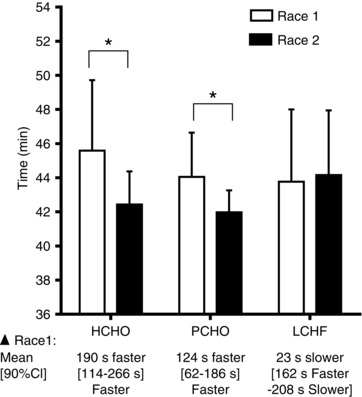
Race times for IAAF sanctioned 10 km race walk events in elite race walkers undertaken pre‐ (Race 1) and post‐ (Race 2) 3 weeks of intensified training and high carbohydrate availability (HCHO, *n* = 9), periodised carbohydrate availability (PCHO, *n* = 8), or ketogenic low carbohydrate, high fat (LCHF, *n* = 9) diets ^*^Significantly different from pre‐treatment (*P* <  0.01).

The participants raced competitively in these events with finishing times for the post‐intervention races representing 103% (4%), 103% (2%) and 108% (9%) of the life‐time personal best times of walkers in the HCHO, PCHO and LCHF groups, respectively. The race times of four race walkers were faster than their personal bests and in one case, represented a national record. Finishing times for the pre‐treatment (Race 1) and post‐treatment (Race 2) are summarised in Fig. 4. The HCHO and PCHO groups completed Race 2 in a significantly faster time than Race 1, showing 6.6% (90% CI: 4.1, 9.1%) and 5.3% (3.4, 7.2%) improvements in performance following the 3‐week diet and training intervention, respectively. Differences in completion times between Race 1 and Race 2 in the LCHF group were not different, and although a practical interpretation of this outcome is an unclear effect on performance (mean change = 1.6% reduction in performance; 8.5% impairment in performance to 5.3% improvement), this reflects an interaction between race conditions and the diet–training treatment.

Capillary blood samples collected pre‐race and post‐race showed a similar increase in blood glucose and lactate concentrations after the race compared with pre‐race values in all groups and races (data not shown). Post‐race measurements of RPE did not differ between groups or races (data not shown). Blood ketone concentrations were low at pre‐ (∼0.1 mmol l^−1^) and post‐race (∼0.3 mmol l^−1^) time points in all groups apart from Race 2 in the LCHF group where they were higher than in the other groups at pre‐ (0.3 mmol l^−1^, 90% CI: 0.03, 0.61) and post‐race (0.73 mmol l^−1^, 90% CI: 0.52, 0.92).

### 25 km long walk

**Figure 5:**
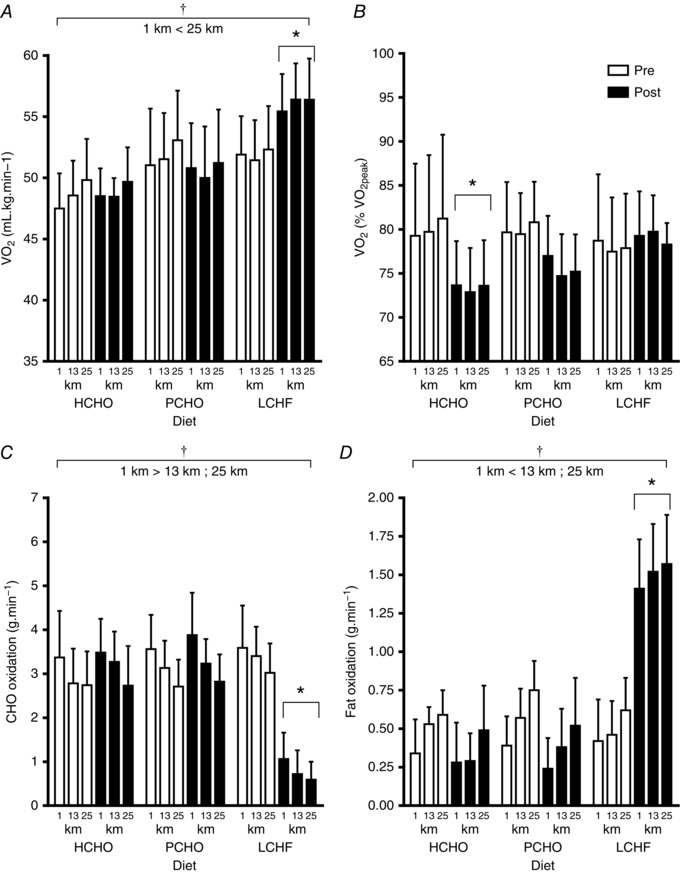
Oxygen uptake (*A*, ml kg^−1^ min^−1^; and *B*, % V˙O2 peak ) and substrate utilisation (*C*, rates of carbohydrate (CHO) oxidation in g min^−1^; and *D*, rates of fat oxidation in g min^−1^) during 25 km standardised long walk in elite race walkers pre‐ and post‐3 weeks of intensified training and high carbohydrate availability (HCHO, *n* = 8), periodised carbohydrate availability (PCHO, *n* = 9) or ketogenic low carbohydrate, high fat (LCHF, *n* = 10) diets ^*^Significantly different from pre‐treatment (*P* <  0.01); †significant change over the 25 km walking session.

Twenty‐seven race walkers completed the long walk before and after the diet and training intervention. Data are missing from two walkers (HCHO and PCHO) who did not participate due to muscle soreness or injury. Therefore, data are presented for *n* = 8, 9 and 10 for the HCHO, PCHO and LCHF groups, respectively. Summaries are provided in Table 5 (BM, HR, RER, RPE and ${\dot{V}}_{O_{2}}$ data), Fig. 4 (${\dot{V}}_{O_{2}}$ and substrate utilisation) and Fig. 5 (blood metabolites). BM decreased 1.18 (90% CI: 0.94, 1.44) kg over the ∼2 h training walks, showing similar levels of dehydration between groups. Overall loss was slightly higher (0.3 kg) in the post‐treatment trials (*F*(1, 23) = 7.05, *P* = 0.01, 90% CI: 0.07, 0.55). At both testing times, there was a decrease in RER across the 25 km walk (*F*(2, 129) = 46.01, *P* < 0.001) associated with a gradual reduction in CHO (*F*(2, 129) = 45.01, *P* < 0.001) and increase in fat oxidation over time (*F*(2, 129) = 33.59, *P* < 0.001) Across the session there was an increase in RPE (*F*(2, 130) = 39.80, *P* < 0.001) and HR (*F*(2, 114) = 10.87, *P* < 0.001) showing an increase in the perceived effort and metabolic cost of exercise. O_2_ utilisation (${\dot{V}}_{O_{2}}$ ml kg^−1^ min^−1^) also gradually increased across the session (*F*(2, 129) = 10.12, *P* < 0.001).

**Table 5:** Results of testing on Day 3: 25 km long walk before and after 3‐week diet and training intervention in elite race walkers (*n* = 29)

		HCHO (*n* = 8)			PCHO (*n* = 9)			LCHF (*n* = 10)		
Variable	Time	1 km	13 km	25 km	1 km	13 km	25 km	1 km	13 km	25 km
BM (kg)	Pre	65.9		64.7	68.0		66.9	68.0		67.0
		(9.2)		(9.3)	(6.9)		(6.6)	(7.4)		(7.5)
	Post	64.5		63.0	66.8		65.3	65.3		63.9
		(8.9)		(8.8)	(6.6)		(6.5)	(7.3)		(7.7)
Respiratory	Pre	0.94	0.90	0.89	0.93	0.90	0.87	0.93	0.92	0.89
exchange ratio		(0.05)	(0.03)	(0.02)	(0.04)	(0.03)	(0.03)	(0.05)	(0.03)	(0.03)
	Post^a^	0.95	0.93	0.89	0.95	0.92	0.89	0.77	0.75	0.74
		(0.05)	(0.02)	(0.04)	(0.04)	(0.03)	(0.03)	(0.04)	(0.03)	(0.03)
${\dot{V}}_{O_{2}}$ (l min^−1^)	Pre	3.14	3.14	3.22	3.44	3.46	3.52	3.52	3.46	3.50
		(0.59)	(0.56)	(0.66)	(0.45)	(0.42)	(0.41)	(0.52)	(0.53)	(0.55)
	Post^b^	3.13	3.09	3.13	3.37	3.30	3.32	3.60	3.63	3.59
		(0.55)	(0.51)	(0.57)	(0.44)	(0.43)	(0.45)	(0.48)	(0.55)	(0.59)
Heart rate (beats min^–1^)	Pre	148	159	160	155	162	167	155	169	167
		(11)	(11)	(11)	(6)	(9)	(7)	(7)	(14)	(7)
	Post†	155	150	153	142	156	152	163	171	169
		(17)	(11)	(11)	(13)	(12)	(7)	(22)	(12)	(12)
RPE	Pre	9.8	13.0	13.8	11.1	12.2	13.8	11.5	12.9	15.3
		(1.6)	(1.7)	(2.0)	(2.4)	(1.4)	(1.4)	(2.4)	(1.8)	(2.8)
	Post‡	11.5	12.3	14.0	11.7	12.2	13.2	12.7	14.5	15.9

Data are mean (SD) and statistical comparisons note differences across the intervention and between groups receiving high carbohydrate availability (HCHO), periodised CHO availability (PCHO) and low carbohydrate high fat (LCHF) dietary treatments. ^a^Significantly lower values in LCHF in post‐treatment trial. ^b^Significantly higher values in LCHF for post‐treatment trial. ^†^Trend for increase in post‐treatment trial in LCHF and trend for decrease in post‐treatment trial in HCHO and PCHO compared with pre‐treatment trial. ^‡^Trend for higher values in LCHF across both trials.

Comparisons between trials and groups (Table 5) showed trends for an increase in HR in the LCHF group in the post‐treatment trial (90% CI: 0.18, 14.25) and a decrease in HR for the HCHO and PCHO groups (−9.81, 0.54 and −12.97, −0.13, respectively). RPE tended to be greater in the LCHF group across both trials (−0.05, 2.18). Examination of the substrate utilisation data shows a significant reduction in RER values (Table 5) in the LCHF group in the post‐treatment trial (−0.20, −0.16) while there was no change from pre‐ to post‐treatment trials in the HCHO and PCHO groups. This finding was accompanied by a concomitant increase in O_2_ utilisation (expressed as ml kg^−1^ min^−1^) in the LCHF post‐treatment trial (Fig. 5). Figure 5 also demonstrates the context of this change in O_2_ utilisation (Fig 5 *A* and *B*); in the case of the HCHO and PCHO groups, the diet‐training intervention was associated with a reduction (−8.44, −5.03), and trend to a reduction (−8.74, −0.49), respectively, in the percentage of maximal aerobic capacity needed to sustain the speed of the training session. However, in the case of the LCHF group, the increase in maximal aerobic capacity achieved over the 3‐week training period was negated by the increased O_2_ cost of walking at the same speed, such that the post‐treatment session was conducted at the same percentage of V˙O2 peak  as the pre‐treatment trial.

Estimation of the rates of CHO and fat oxidation in the 25 km walk (Fig. 5 *C* and *D*) showed a decrease in CHO oxidation and a concomitant increase in fat oxidation over the session (*F*(2, 129) = 45.01, *P* ≤ 0.001 and *F*(2, 129) = 33.59, *P* < 0.001, respectively). However, substrate utilisation was dramatically changed in the post‐treatment trial for the LCHF group, with a large reduction in CHO oxidation rates (90% CI: −2.98, −2.47) and increase in rates of fat oxidation (1.03, 1.23).

**Figure 6:**
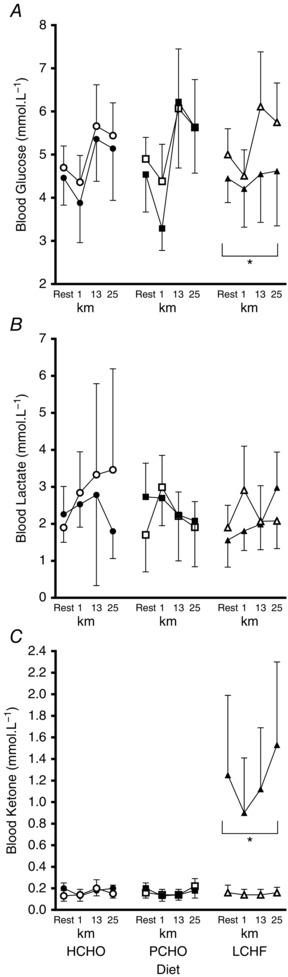
Blood metabolite concentrations (blood glucose (mmol l^−1^, *A*), blood lactate (mmol l^−1^, *B*) and blood ketones (β‐hydroxybutyrate, mmol l^−1^, *C*) during 25 km standardised long walk in elite race walkers pre‐ and post‐3 weeks of intensified training and high carbohydrate availability (HCHO, *n* = 8), periodised carbohydrate availability (PCHO, *n* = 9), or ketogenic low carbohydrate, high fat (LCHF, *n* = 10) diets ^*^Significantly different from pre‐treatment (*P* <  0.01).

Figure 6 shows metabolites in capillary blood samples collected immediately prior to and during the 25 km walk session, which was undertaken post‐meal and with nutritional support according to dietary treatment. There was a small decrease in blood glucose concentrations (Fig. 6 *A*) during the first 5 min of the session prior to the intake of CHO; thereafter, blood glucose concentrations were increased at 13 km (90% CI: 0.11, 1.53) followed by a slight non‐significant decline at 25 km (−0.13, 1.27). There was a time by treatment interaction whereby blood glucose concentrations were maintained at lower concentrations throughout the 25 km walk on the LCHF post‐treatment trial compared with the pre‐treatment trial and the other groups (*F*(6, 167) = 2.25, *P* = 0.04, 90% CI: −2.71, −0.18). Blood lactate concentrations (Fig. 6 *B*) were maintained (< 4 mmol l^−1^) across the exercise session in all trials. Blood ketone concentrations (Fig. 6 *C*) were maintained at low concentrations (< 0.2 mmol l^−1^) across the exercise session in all trials, except for the post‐treatment trial in the LCHF group where they were significantly elevated (0.78, 1.31) and maintained at ∼1 mmol l^−1^ across the session.

## Discussion

This is the first study of the effect of a ketogenic low‐CHO, high fat diet consumed during a period of intensified training in elite athletes in which exercise characteristics across the range of intensities at which endurance and ultra‐endurance athletes train and compete were investigated. The novel findings were as follows. (1) Three weeks of intensified training and mild energy deficit in already well‐trained athletes increased peak aerobic capacity, independent of dietary support. (2) An LCHF diet was associated with the highest rates of whole‐body fat oxidation ever reported across exercise of varying speeds or intensities. (3) The cost of a shift in substrate oxidation (from CHO‐based to fat‐based fuels) during exercise was an increased oxygen demand for a given walking speed across velocities that translate to real‐life race performance in elite race walkers. (4) An improvement in performance was achieved by training and competing while consuming diets providing periodic or chronic high CHO availability, but not with the LCHF diet.

Our study was conducted over an ∼4‐week period, which included a 3‐week period of intensified training. Previous experience of undertaking training studies in well‐trained but lesser calibre endurance athletes (Lindsay *et al*. 1996; Yeo *et al*. 2008a; Cox *et al*. 2010) has shown this to be a sufficient time frame to induce detectable perturbations in muscle metabolism and performance. The exposure to the LCHF diet in the current investigation comfortably exceeded the time frame shown to produce robust cellular adaptations to ‘retool’ the muscle to increase its capacity for fat oxidation (Burke *et al*. 2000, 2002; Carey *et al*. 2001; Cameron‐Smith *et al*. 2003). For example, in the case of a non‐ketogenic high fat diet, peak rates of fat oxidation occur in as little as 5 days (Goedecke *et al*. 1999). However, in the case of the ketogenic LCHF diet, where further adaptation occurs to allow near‐excusive reliance on fat‐derived fuels, it has been suggested that several weeks of exposure is required before the fatigue and general loss of well‐being associated with such an extreme dietary shift abates (Phinney *et al*. 1983). With regard to the periodisation of CHO availability, we have recently shown in well‐trained but sub‐elite triathletes that a 3‐week intervention of this type was able to produce performance improvements compared with a more traditional diet of sustained high CHO availability (Marquet *et al*. 2016). The current study was limited to a tightly controlled intervention and rigorously collected non‐invasive measures of whole‐body metabolism and performance to allow us to work with elite athletes in a training camp environment with field and laboratory activities. Collectively, these factors give our results ‘real‐world’ credibility while also offering valuable insights into this topical area of exercise science.

Our data show that the LCHF group experienced changes in blood metabolites and substrate utilisation as a result of their dietary treatment. There was a general decrease in blood glucose concentrations at rest and during exercise, while blood ketone (β‐hydroxybutyrate) concentrations were generally elevated within the range of 0.8–2.0 mmol l^−1^. Respiratory gas exchange measurements showed a substantial increase in whole‐body rates of fat oxidation and a concomitant reduction in CHO utilisation during the graded economy test across the range of walking speeds, as well as the sustained intensity 25 km training session. Although we did not investigate the mechanism(s) underpinning the increases in utilisation of fat during exercise, previous studies on high fat diets from our group and others have reported changes including increases in intramuscular triglyceride (Yeo *et al*. 2008b), an increase in hormone‐sensitive lipase (Stellingwerff *et al*. 2006), increases in the expression of fatty acid translocase FAT/CD36 protein (Cameron‐Smith *et al*. 2003) and carnitine palmitoyl transferase (Goedecke *et al*. 1999). Collectively, these changes suggest increases in fat availability, mobilisation and transport activities within the complex regulation of fat utilisation by the muscle.

Rates of fat oxidation were calculated during the 25 km training session to allow comparison with results from other studies of adaptation to low‐CHO, high fat diets. Our previous research on short term (5 days) adaptation to a non‐ketogenic low(er) carbohydrate (< 20% of energy), high(er) fat (65% of energy) diet found whole‐body rates of fat oxidation of ∼60 and ∼70 μmol kg^−1^ min^−1^ in well‐trained cyclists during training sessions at 85% and 65% of V˙O2 peak , respectively (Stepto *et al*. 2002). These figures approximate to absolute rates of fat oxidation of ∼1.3–1.5 g min^−1^ and represented a doubling of fat utilisation in these cyclists compared with their substrate use at the same exercise intensity on a high CHO diet. Meanwhile, in the only other study involving the ketogenic LCHF diet in endurance athletes (Phinney *et al*. 1983), cyclists who were exposed to 4 weeks of a ketogenic (< 20 g day^−1^ CHO) high fat (85% of energy) diet showed mean rates of fat oxidation of ∼1.5 g min^−1^ while cycling at 62–64% V˙O2 max  following an overnight fast. Other available data on the ketogenic LCHF diet come from recently published cross‐sectional studies of ultra‐endurance athletes who have self‐selected and self‐reported long‐term (> 6 months) adherence to this dietary strategy. In one investigation (Webster *et al*. 2016), cyclists habituated for ∼13 months to an LCHF diet (< 50 g day^−1^ or 10% of energy CHO; ∼70% of energy from fat) sustained mean rates of fat oxidation of 1.2 g min^−1^ during 2 h of cycling at ∼70% V˙O2 max  compared with a gradual drift to 0.5 g min^−1^ by a matched group of athletes who consumed diets providing higher CHO availability. However, Volek and co‐workers (2016) reported the highest peak rates of fat oxidation in the literature in elite ultra‐distance triathletes/runners with a mean adherence of 20 months to LCHF nutrition (∼10% energy from CHO, ∼70% energy from fat). These rates (1.54 ± 0.18 *vs*. 0.67 ± 0.14 g min^−1^ occurring at ∼70% *vs*. 55% V˙O2 max ) were measured during a graded protocol involving short (2 min) exercise periods and attributed to the duration of the adherence to the LCHF diet. These same athletes were found to have fat oxidation rates of 1.21 *vs*. 0.76 g min^−1^ over 3 h of submaximal treadmill running at 64% V˙O2 max , following a pre‐exercise meal based on their habitual dietary intake (Volek *et al*. 2016). In the current study, we observed sustained rates of fat oxidation of ∼1.5 g min^−1^ in our elite race walkers, reaching a peak of 1.57 ± 0.32 g min^−1^ towards the end of 2 h of exercise undertaken at ∼80% V˙O2 peak  (50 km race pace). These rates represent a 2.5‐fold increase on the pre‐treatment values of 0.62 ± 0.32 g min^−1^. In some individuals we observed peak fat oxidation rates exceeding 1.9 g min^−1^, and note they were achieved by adaptation to the LCHF diet for only 3 weeks, in concert with a high fat pre‐exercise meal and further fat intake during the session. Our findings confirm that remarkable alterations in substrate utilisation can be achieved in well‐trained athletes; indeed, to the best of our knowledge, these are the highest rates of fat oxidation reported. Moreover, they may address the potential criticism that the current study was too brief since it appears to have achieved the most hallmarked feature of athletes who have ‘keto‐adapted’ for much longer periods.

Concomitant with the increased rates of fat oxidation, there was a decrease in rates of CHO oxidation in the LCHF group, as reported previously in studies in which CHO intake was substantially reduced (Goedecke *et al*. 1999; Burke *et al*. 2000; 2002; Carey *et al*. 2001), or restricted (Phinney *et al*. 1983; Volek *et al*. 2016; Webster *et al*. 2016). Mechanisms for the down‐regulation of CHO metabolism include the reduced availability of CHO substrate (e.g. reduced muscle glycogen stores, lower plasma glucose concentrations and the absence of exogenous intake of CHO during exercise). However, we have previously found, at least in the case of adaptation to a non‐ketogenic low CHO diet, a reduction in glycogenolysis during exercise, and a reduction in the active form of pyruvate dehydrogenase (PDHa) at rest and during exercise of both moderate‐ and supra‐maximal exercise, thus reducing the capacity for an oxidative fate of CHO disposal even when the supply is adequate (Stellingwerff *et al*. 2006). The reliance on measurements of whole‐body metabolism in the current study prevents a more mechanistic investigation of the changes in CHO storage and utilisation. However, the maintenance of blood glucose concentrations, albeit at reduced levels, and the increases in blood lactate concentrations during the graded economy and aerobic capacity tests indicate the presence of endogenous CHO stores despite minimal CHO intake. Indeed, due to the importance of glucose as a substrate for many tissues and a source of carbon for biosynthesis and anaplerosis, humans can adapt to conditions of food or CHO deprivation by synthesising glucose from a variety of substrates and reducing CHO oxidation (Soeters *et al*. 2012).

While one study of ultra‐endurance athletes habituated to an LCHF diet found little difference between that and a high‐CHO diet with regard to the storage and subsequent utilisation of muscle glycogen while running (despite paradoxical reductions in the rates of CHO oxidation during exercise) (Volek *et al*. 2016), the majority of observations of keto‐adapted athletes have reported modest reductions in resting muscle glycogen stores and lower rates of utilisation during exercise (Phinney *et al* 1983; Webster *et al*. 2016). Tracer technology and muscle biopsies allowed Phinney and co‐workers (1983) to estimate that glycogen utilisation was reduced 4‐fold, and blood glucose utilisation, 3‐fold, during moderate intensity (62–64% V˙O2 max ) cycling when cyclists adapted to an LCHF diet, and they hypothesised that gluconeogenesis (from glycerol, lactate, pyruvate and gluconeogenic amino acids) was able to maintain blood glucose concentrations and permit glycogen restoration during recovery from exercise. Webster *et al*. (2016) measured glucose kinetics at rest and during 2 h of cycling at a slightly higher intensity (∼70% V˙O2 peak ) and showed that endogenous glucose production at rest and during exercise in keto‐adapted ultra‐endurance athletes was lower compared with athletes consuming higher CHO diets. They hypothesised that this reduced endogenous glucose production was due to a reduction in liver glycogen breakdown that was not compensated for by an absolute increase in gluconeogenesis (Webster *et al*. 2016) while noting that although absolute rates of gluconeogenesis were similar to those seen in fasted athletes with a habitually high CHO intake, they now contributed a greater proportion of body CHO stores, and possibly came from different substrates (glycerol, preferentially in the case of the LCHF group and lactate in the case of the high CHO group). In the current study we can only speculate on the exact nature of changes in CHO utilisation and storage with the LCHF diet. Although we recognise that plasma metabolite concentrations can mask changes in the rates of appearance and disappearance, high plasma lactate concentrations were observed after the 10 km race and the test of peak aerobic capacity, suggesting that the glycolytic fate of body CHO stores was preserved in the face of significant reductions in CHO oxidation.

In the present study, all groups of race walkers improved their aerobic capacity by 3–7% following the intensified training programme, independent of dietary intervention. Despite this, and their enhanced capacity to oxidise fat during higher intensity exercise, the race walkers in the LCHF diet failed to gain the improvement in 10 km race performance achieved by the HCHO and PCHO groups. This is a crucial finding for both competitive athletes and sports scientists, and the mechanisms underpinning the impairment of performance merit further discussion. Other factors such as increased perception of effort associated with exercise or effects of the reduced quality of workouts during the training programme may have contributed to the failure of the LCHF group to improve race performance, but the effect of the LCHF on the economy of race walking at speeds required for competitive performance is of major interest. The increased oxygen cost of ATP production from fat *vs*. CHO oxidation is self‐evident from stoichiometry and has been known empirically for a century (Zuntz & Schumburg 1901; Krogh & Lindhard, 1920). Indeed, since the oxidative phosphorylation yield is higher when NADH is the electron donor (3 coupling sites) compared with FADH_2_ (2 coupling sides), and CHO metabolism produces a greater ratio of the reducing equivalents NADH/FADH2 than β‐oxidation, CHO is able to produce a greater ATP yield per unit of oxygen consumption despite the greater ATP production per unit of substrate from fat (Leverve *et al*. 2007). However, to the best of our knowledge, this notion appears to have been largely ignored in the recent discussions regarding LCHF and sports performance.

In sports such as running, cycling, swimming and race walking, the economy or efficiency of energy transfer to the speed of movement is a key determinant of performance (Joyner & Coyle, 2008). For example, although many elite runners present with a high aerobic capacity, within a homogeneous group, running economy (defined as the relationship between oxygen consumption and running velocity) at specific race paces is better correlated to performance than V˙O2 peak  (Daniels & Daniels, 1992). In the present study, the oxygen cost of race walking at speeds approximating race pace in both 20 km events and the 50 km event was significantly increased with the LCHF intervention. Indeed, in the LCHF group, this negated the benefit of the training‐induced increase in peak aerobic capacity. In contrast, the athletes training and competing with high CHO availability achieved a reduction in the relative oxygen cost of walking, notably at the higher velocity of the shorter event on their race programmes. Others have also shown that the energy expenditure of cycling is reduced, and gross efficiency increased, following short‐term consumption of a high‐CHO compared with a low‐CHO diet (Cole *et al*. 2014). When exercise is undertaken at a modest fractional utilisation of an athlete's aerobic capacity, there is opportunity to compensate for a loss of economy by increasing oxygen uptake. However, during activities conducted at or around an individual's lactate threshold, such compensation is not possible and reduced ATP production from the available oxygen supply will limit exercise capacity.

The previous studies of LCHF in trained populations (Phinney *et al*. 1983; Volek *et al*. 2016; Webster *et al*. 2016) have examined exercise capacity at moderate exercise intensities (60–70% V˙O2 max ) that are unrelated to race pace in competitive endurance athletes (Hawley & Leckey 2015). We have previously noted that the discussion around LCHF and sports performance in both lay and scientific media often shows a poor understanding of the exercise characteristics that are important to competitive athletes (Burke, 2015; Hawley & Leckey 2015). For example, sports such as multi‐stage road cycling, triathlons and marathons are often described as endurance and ultra‐endurance events conducted at sub‐maximal exercise intensities; in fact, for competitive athletes at least, the terrain, pacing strategies and tactical elements in these events mean that brief but critical parts of the race, which often determine the outcomes (e.g. breakaways, hill climbs, surges, sprint finishes), are conducted at higher and often near maximal pace (Fernandez‐Garcia *et al*. 2000; Bentley *et al*. 2002; Tucker, 2016 2016). In addition, for many athletes, even the ‘background’ pace from which these brief bursts are performed in endurance sports such as the marathon requires high exercise economy and a sustained use of a very high percentage of maximal aerobic intensity (Joyner *et al*. 2011; Tucker, 2016 2016). The V˙O2 peak  values (65–75 ml kg^−1^ min^−1^) measured in our elite race walkers are not exceptionally high in relation to values often cited for elite endurance runners and cyclists, although consideration must be given to the time of the season, the race walking test protocol and moderate altitude of 600 m of the testing facility. Nevertheless, they are comparable to values reported in other studies of international level male race walkers (Chwała *et al*. 2014) and we note that our group included several of the world's best athletes in this Olympic sport. Importantly, our studies show that performance in their events requires them to compete (and undertake substantial amounts of training) at a high percentage of their aerobic capacity where CHO oxidation is the major fuel‐generating pathway (Hawley & Leckey, 2015).

The 20 km race walk requires mean velocities of 15.6 and 14.3 km h^−1^ to achieve the world record of 76:36 min and qualifying standard for the 2016 Rio Olympics of 84 min, respectively. Indeed, even after the period of intensified training, which improved the relative economy of the HCHO and PCHO groups, walking at a velocity approximating an individual's personal best race pace for the 20 km event within the graded economy protocol required a fractional oxygen utilisation of ∼85–90% V˙O2 peak . The corresponding data for the 50 km event (world record of 3:32.33 (h:min.s) and Olympic qualifying standard of 4:06 h are a mean race pace of 14.1 and 12.2 km h^−1^, respectively. Among our participants, walking at a velocity of 12–13 km h^−1^ to approximate the 50 km race pace of at least the middle calibre athletes in our group required ∼75% of their increased post‐training peak aerobic capacity in the graded economy test and 25 km standardised walk for the higher CHO groups. However, it remained at a mean of ∼80% V˙O2 peak  of the LCHF group in the post‐treatment walk. It should also be noted that the mean 50 km race pace for the top competitors in our group is higher than the stage of the economy test at which we recorded these results and therefore mean race pace is conducted at a higher percentage of peak aerobic capacity, and key parts of the race at even faster paces.

Other sources support the concept that adaptation to a high fat diet impairs capacity/performance of sustained higher intensity exercise via a down‐regulation of CHO oxidation. Indeed, Phinney *et al*. (1983) noted that although chronic adaptation to an LCHF diet improved fat oxidation to the point that capacity for moderate submaximal exercise was ‘comparable to that observed after a high CHO diet’, ‘the price paid for such extreme conservation of CHO during exercise appears to be a limitation on the intensity of exercise that can be performed’. Havemann and colleagues (2006) found that a 5 day fat‐adaptation followed by restoration of CHO availability on the day before and during a performance permitted similar overall performance of a 100 km cycling time‐trial compared with a trial undertaken following a chronic high CHO availability approach, including similar performances of 4 km sprints (∼80% peak power output) interspersed within the time trial. However, when required to undertake 1 km sprints at > 90% of peak power output (∼95% of V˙O2 max ) within the time‐trial, there was a significant decrease in power output despite the maintenance of perceived effort and heart rate and an increased attempt to recruit muscle fibres (Havemann *et al*. 2006).

Another factor that has contributed to confusion about the LCHF diet and sports performance is a misunderstanding of the goal and outcomes of endurance training. An increase in fat utilisation during exercise is the most frequently discussed outcome of endurance training in relation to muscle fuel (Holloszy & Coyle 1984). While there is a training‐induced increase in rates of fat oxidation during exercise undertaken at the same *absolute* intensity, it is perhaps not well appreciated that the fractional contribution of fat to the new (higher) workload corresponding to the same *relative intensity* of the athlete's increased peak aerobic capacity remains essentially the same (Bergman & Brooks, 1999). Indeed, another goal of endurance training is to increases an athlete's absolute capacity to use CHO as a fuel substrate, via both oxidative and oxygen‐independent pathways (Hawley & Leckie, 2015), and a more holistic understanding of the ideal training programme is that it should enhance an athlete's capacity to utilise and integrate all of the body's range of energy‐producing pathways, with special focus on ways in which they become specifically limiting for performance.

The training intervention in the current study is representative of the periodised programme undertaken by endurance athletes to include a variety of workouts with different goals within a training phase. We attempted to expand on this principle, by including a separate arm of the study in which we manipulated CHO intake around specific training sessions to better achieve the adaptation and performance goals of the workout. For example, within the weekly training cycle we matched sessions featuring moderate intensity ‘recovery’ work with practices such as training in a fasted state or training with low glycogen concentrations to promote mitochondrial biogenesis and up‐regulation of fat oxidation (for review, see Bartlett *et al*. 2015). We also coupled practices to enhance CHO availability with sessions requiring good performance of higher intensity exercise as well as opportunities to practice race day CHO intake and to increase capacity for gut tolerance and absorption of CHO (Stellingwerff, 2013). However unlike the recent study in which we undertook such a manipulation in a 3‐week training programme with well‐trained triathletes (Marquet *et al*. 2016), we failed to see a benefit of this CHO periodisation over the intervention in which all sessions were undertaken with strategies aimed at achieving high CHO availability. It is uncertain whether the lack of additional benefit relates to the calibre of our athletes, the involvement of our intervention in the base phase of the training programme (where the increased training stimulus may have already maximised the adaptive response and reduced the capacity for differences due to CHO availability) or the large training volumes (which might have depleted CHO stores even in the face of the high CHO intakes around training such that the actual differential between the HCHO and PCHO interventions was reduced).

There are a number of other elements raised in this study that warrant further investigation. These include a titration of exercise intensity and exercise economy to find the pace at which the negative effects of the LCHF on exercise economy become detectable, thus differentiating the real‐life sporting events and athletes for which this represents an unsuitable practice. The potential for keto‐adaptation as a periodised rather than chronic activity, and the time course of some elements of the ‘de‐adaptation’ should also be considered. The effect of a low carbohydrate intake on gut absorption of CHO is worthy of merit since it is likely to be reduced, in the same way that an increased intake can improve the capacity for muscle use of exogenous CHO sources consumed during long exercise (Cox *et al*. 2010). This is of importance to the longer endurance events in which CHO intake during the race provides a substantial fuel source and is associated with enhanced performance (Stellingwerff & Cox, 2014), and is relevant since popular advice to athletes contemplating the use of LCHF practices includes the possibility that some may still choose to consume CHO during their competitive events. The role of ketones as a muscle fuel source was not investigated in the current study but is also of interest (Cox *et al*. 2016).

In conclusion, the results of the present study showed that despite achieving substantial increases in the capacity for fat oxidation during intense exercise, chronic adaptation to a ketogenic low‐CHO, high fat diet impaired exercise economy and negated the transfer of training‐induced increases in aerobic capacity into improved performance of a real‐life endurance event in elite athletes. In contrast, training with a diet rich in carbohydrate and which provided either high or periodised carbohydrate availability around training sessions was associated with improved race outcomes.

## Additional information

### Competing interests

None of the authors of this paper has a competing interest.

### Author contributions

This study was conducted at the Australian Institute of Sport, Canberra, Australia. Conception and design of the experiments was undertaken by L.M.B., M.L.R., L.A.G.‐L., M.W., I.A.H., S.G.F., J.G.M., L.E.C., N.S., A.P.S. and J.A.H. Collection, assembly, analysis and interpretation of data was undertaken by L.M.B., M.L.R., L.A.G.‐L., M.W., I.A.H., S.G.F., J.G.M., L.E.C., N.S. and A.P.S. Drafting the article or revising it critically for important intellectual content was undertaken by L.M.B., M.L.R., L.A.G.‐L., M.W., I.A.H., S.G.F., J.G.M., L.E.C., N.S., A.P.S. and J.A.H. All authors have approved the final version of the manuscript and agree to be accountable for all aspects of the work. All persons designated as authors qualify for authorship, and all those who qualify for authorship are listed.

### Funding

This study was funded by a Program Grant from Australian Catholic University Research Fund (No. 201300800) and a grant from the Australian Institute of Sport's High Performance Sport Research Fund.
